# The role of chemistry in the success of oligonucleotides as therapeutics

**DOI:** 10.3762/bjoc.18.22

**Published:** 2022-02-14

**Authors:** Pawan Kumar, Tom Brown

**Affiliations:** 1 Takeda Development Center Americas, Inc. (TDCA), 9625 Town Centre Drive, San Diego, CA 92121, USA; 2 Chemistry Research Laboratory, University of Oxford, 12 Mansfield Road, Oxford, OX1 3TA, UK

**Keywords:** antisense oligonucleotides, chemically modified nucleotides, siRNAs

RNA-targeting oligonucleotides (e.g., antisense, siRNA, and anti-miR) are widely explored as fundamental research tools and are gaining increasing promise as therapeutic agents, particularly against diseases of genetic origin. The idea of treating a disease by targeting the molecular messenger (mRNA) to stop the synthesis of proteins using short strands of DNA, now known as antisense oligonucleotides, was first coined about 40 years ago [1]. Almost 20 years later, another endogenous mechanism, known as RNA interference (RNAi) was discovered when it was shown that short stretches of double-stranded nucleotides, which are called short interfering RNA or “siRNA,” can target mRNA and prevent it from being translated to make proteins [2]. While the mechanism by which antisense oligonucleotides (single stranded oligonucleotide) and siRNA (short RNA duplexes) work are completely different, both of them target mRNA to disrupt protein synthesis. In the following text we will refer both antisense oligonucleotides and siRNAs collectively as therapeutic oligonucleotides.

**Figure 1:**
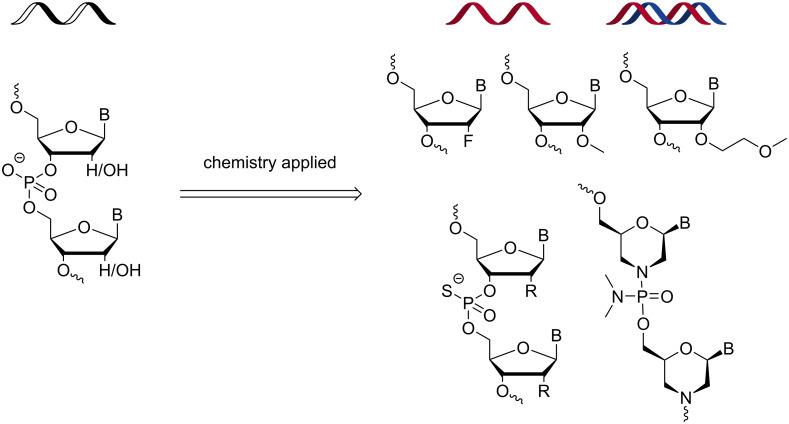
Introduction of chemically modified nucleotides into oligonucleotides is an essential part of their development as therapeutics.

More than 10 oligonucleotide drugs have received regulatory approval by the FDA and are now helping patients suffering from conditions that were previously seen as untreatable. However, the road from bench to bedside for therapeutic oligonucleotides has not been straightforward. It took about four decades (from discovery of the antisense mechanism) to reach the point where it is now possible to treat previously undruggable conditions using therapeutic oligonucleotides. There were many challenges including, but not limited to, poor stability of unmodified DNA strands and short RNA duplexes in cells, large anionic charge, poor drug-like properties, and a tendency to trigger the immune response in the body. Chemists have been at forefront of solving these issues, and have introduced many chemically modified nucleotides into oligonucleotides to increase their binding affinity toward RNA targets, and to improve their stability against nucleases to slow down degradation. This strategy has been successful, and most oligonucleotide-based drugs that have been approved by the FDA contain chemically modified nucleotides (Figure 1) indicating the critical role chemists have played in bring oligonucleotides from bench to bedside. Importantly, a plethora of different chemically modified nucleotides have been described in the literature [3], and the field owes a big thanks to all the researchers who have contributed to expanding chemical space of modified nucleotides (building blocks of oligonucleotides). However, discussing these modifications in detail is beyond the scope of this editorial.

The use of chemically modified nucleotides massively improves the drug-like properties of oligonucleotides. However, their efficient delivery to the desired tissue/organ also needs to be addressed. Conjugation of oligonucleotides to GalNAc (*N*-acetylgalactosamine) has successfully been used for targeted delivery of oligonucleotides (both antisense and siRNAs) to the liver. Currently, there are three siRNA-GalNAc conjugates approved by the FDA and many others are in late-stage clinical trials. The success of GalNAc-oligonucleotide conjugates highlights the power of therapeutic oligonucleotides in treating previously untreatable conditions, once a means to deliver them into a desired tissue has been achieved. However, challenges remain in finding suitable GalNAc equivalents for delivering oligonucleotides to tissues other than the liver. Delivery of oligonucleotides to extra hepatic tissues is certainly an area where many academic and biotech laboratories are focused. In this context antibody-oligonucleotide conjugates have shown promise in targeted delivery and are entering into clinical trials. Chemistry is at the forefront of discovering new conjugates/modifications and it is hoped that solutions will be found to address the huge unmet medical need in the CNS space, and to treat diseases that have been elusive until now.

Developing lipid nanoparticles that can selectively deliver oligonucleotides to a desired tissue is also an attractive strategy. Importantly, siRNA encapsulated into lipid nanoparticles has been shown to be effective in patisiran, the first RNAi drug to reach patients. Lipid nanoparticles are also being used in the new generation of RNA vaccines for tackling the COVID pandemic.

Another noteworthy advancement is the ease and scale with which oligonucleotides are being produced today. Without access to larger quantities of oligonucleotides it would not have been possible to develop them as therapeutic agents. Forty years ago, the synthesis of an oligonucleotide in the lab was a huge task. Since then, seminal work from the Caruthers lab has solved this problem with the introduction of phosphoramidite chemistry [4]. This proved an outstanding breakthrough and is currently catalyzing the development of many new technologies including next generation siRNAs, antisense oligonucleotides, and CRISPR-based gene editing systems. Thanks to this phosphoramidite approach, it has also been possible to mass-produce oligonucleotide primers and probes for use in diagnostic testing kits for the detection of COVID-19 for tackling the pandemic.

With this thematic issue, we express our sincere gratitude to all the scientists for their ground-breaking work to bring oligonucleotide therapeutics to the bedside. We also express our sincere thanks to the authors and reviewers who have contributed despite the challenges posed by the COVID pandemic. Support from the editorial team of the *Beilstein Journal of Organic Chemistry* is also greatly appreciated. The issue comprises many excellent contributions in the form of original research and review articles from world-leading experts in the field. We hope the thematic issue will inspire readers and provide a state of the art background on emerging areas in the rapidly evolving therapeutic oligonucleotide field.

Pawan Kumar and Tom Brown

San Diego and Oxford, January 2022
